# Emerging avenues in immunotherapy for the management of malignant pleural mesothelioma

**DOI:** 10.1186/s12890-021-01513-7

**Published:** 2021-05-05

**Authors:** Steven G. Gray

**Affiliations:** 1 Thoracic Oncology Research Group, Central Pathology Laboratory, CPL 30, TCDSJ Cancer Institute, St James’s Hospital, Dublin, D08 RX0X Ireland; 2 Department of Clinical Medicine, Trinity College Dublin, Dublin, Ireland; 3 School of Biology, Technical University of Dublin, Dublin, Ireland

## Abstract

**Background:**

The role of immunotherapy in cancer is now well-established, and therapeutic options such as checkpoint inhibitors are increasingly being approved in many cancers such as non-small cell lung cancer (NSCLC). Malignant pleural mesothelioma (MPM) is a rare orphan disease associated with prior exposure to asbestos, with a dismal prognosis. Evidence from clinical trials of checkpoint inhibitors in this rare disease, suggest that such therapies may play a role as a treatment option for a proportion of patients with this cancer.

**Main text:**

While the majority of studies currently focus on the established checkpoint inhibitors (CTLA4 and PD1/PDL1), there are many other potential checkpoints that could also be targeted. In this review I provide a synopsis of current clinical trials of immunotherapies in MPM, explore potential candidate new avenues that may become future targets for immunotherapy and discuss aspects of immunotherapy that may affect the clinical outcomes of such therapies in this cancer.

**Conclusions:**

The current situation regarding checkpoint inhibitors in the management of MPM whilst encouraging, despite impressive durable responses, immune checkpoint inhibitors do not provide a long-term benefit to the majority of patients with cancer. Additional studies are therefore required to further delineate and improve our understanding of both checkpoint inhibitors and the immune system in MPM. Moreover, many new potential checkpoints have yet to be studied for their therapeutic potential in MPM. All these plus the existing checkpoint inhibitors will require the development of new biomarkers for patient stratification, response and also for predicting or monitoring the emergence of resistance to these agents in MPM patients. Other potential therapeutic avenues such CAR-T therapy or treatments like oncolytic viruses or agents that target the interferon pathway designed to recruit more immune cells to the tumor also hold great promise in this hard to treat cancer.

## Background

MPM is an aggressive inflammatory cancer associated with exposure to asbestos. Despite having been banned in the western world, current data from the US has shown that the rate of MPM in males has remained constant from 1994, while the rate of MPM in females has remained unchanged for decades [1]. Indeed, while the use of asbestos has declined in industrialized nations, asbestos is still being exported to developing nations [2, 3]. Moreover, environmental exposure is still widespread due to (a) previous industrial use; (b) its difficulty to remove; (c) natural deposits are being disturbed by human activities; and (d) housing proximity to these natural deposits [1, 4–7].

The economic burden for MPM is significant both at the level of total cost for hospital care [8, 9], and economic burden [9, 10].

Clinically, if untreated, MPM has a median survival time of 6 months, and most patients die within 24 months of diagnosis. The current standard of care (SOC) is a combination of pemetrexed/raltitrexed and cisplatin chemotherapy) [11] is non-curative and results in a response rate of ∼ 40% [12], and there is no standard second line therapy once treatment fails. Recently, the addition of an anti- vascular endothelial growth factor (anti-VEGF) therapy (Bevacizumab) has been shown to enhance OS when given in the first line setting [13]. And whilst this therapeutic combination is now the new standard of care in France [14], it has not yet been approved by the FDA, issues with cost and lack of reimbursement prevent it from being added to the SOC in many countries, and other anti-angiogenic combinations have not been successful [15].

The power of the human immune system to prevent cancer (often described as immune-surveillance) was first mooted by Ehrlich in 1909 [16, 17]. One of the mechanisms used by cancer cells to evade immune surveillance involves a series of surface regulatory markers (called checkpoint molecules), and has led to the development of checkpoint inhibitors for cancer therapy, an area of active investigation in MPM. Other prominent new treatment options emerging in MPM (and other cancers) involve cancer immunotherapy, a situation where the patient's own immune system (antibodies, cells, cytokines, etc.) is exploited to eliminate tumor cells [17, 18]. In the following review we examine some of the current clinical studies of immunotherapies in mesothelioma, explore some of the issues potentially linked to lack of objective responses, and discuss alternative immunotherapy targets which may translate into mesothelioma clinical trials moving forwards.

## Immunotherapy in MPM in the historical setting

Historically, immunotherapy in mesothelioma is not new, and studies involving this cancer have been attempted for over 25 years [19]. Examples of early trials in this arena predominantly used Interleukin-2 (IL-2) and Tumor Necrosis Factor alpha (TNF-α), were ineffective and suffered particularly from a lack of scalability and logistical issues [19, 20]. Some encouraging clinical responses were observed for patients with good performance status [21] while more recent studies in animal models suggest that direct injection of IL-2 plus an agonist anti-CD40 antibody induces regression of large mesothelioma tumors through a mechanism involving natural killer (NK) cells driven acquisition and/or maintenance of systemic immunity and long-term effector/memory anti-mesothelioma responses [22].

Some of the earliest trials involved the infusion of interferon (IFN) gamma to treat malignant pleural effusions [23, 24], oftentimes with complete responses in Stage I patients [24]. Follow up studies using intra-pleural infusion of interferon-gamma in a larger cohort of (n-89) patients observed a 20% overall response rate with most responses in early stage disease especially if the tumor was confined to the parietal or diaphragmatic pleura [25]. Whilst these and other studies of interferon therapy combined with chemotherapy regimens suggest that this strategy could be useful [26, 27] with median survival rates of approximately 8–12 months, other studies found significant toxicities [28]. Later studies using intrapleurally infused autologous human activated macrophages combined with interferon gamma found limited antitumor activity [29], while a study involving debulking surgery coupled with interferon based immunotherapy also demonstrated limited overall survival benefit [30], suggesting that interferon therapy has limited clinical benefit in MPM.

Another potential immunotherapy target for MPM involves Granulocyte macrophage colony-stimulating factor or GM-CSF, used as an immune-stimulatory adjuvant to elicit antitumor immunity [31, 32]. Initial studies in MPM involved infusions of GM-CSF [33–35] but few or no responses were observed [34, 35], and high toxicity [33] or a poor OS (median survival of 7 months) were the outcomes. A small clinical trial (n = 22 patients) was conducted involving a vaccination strategy comprising autologous mesothelioma tumor cell lysate combined with GM-CSF was conducted. The trial was found to be safe, and induced tumor specific immunity in 32% of patients, but saw only stable disease ad no tumor objective responses [36]. More recently, tumor derived GM-CSF was shown to actually promote immunosuppression in mesothelioma suggesting that actually targeting this molecule may be more effective in augmenting immunotherapy in MPM [37].

### Checkpoint inhibitor immunotherapy within the neo-adjuvant setting

Although not SOC, there is compelling evidence that a select subgroup of mesothelioma patients benefit from a surgery-based multimodal approach, particularly if they have an epithelioid histological subtype, lower-volume disease, and/or minimal to no nodal involvement [38]. In MPM microscopic complete resection is considered to not be achievable, and patients who have surgically resectable disease often undergo an aggressive multi-modality therapy for which the optimal combination therapy has not yet been identified [39]. Various taskforces have been set to explore the various options, and some proposed consensus reports have recently been published [40–42]. In this regard neoadjuvant immunotherapy prior to surgery has been mooted as an advantageous prospect in the management of solid tumors as they enhance T-cell activation the moment antigen is encountered, and encouraging findings from early-phase clinical trials in various cancers support this notion [43–45]. A series of Phase I/II clinical trials involving neo-adjuvant immunotherapy prior to surgical resection have been initiated in MPM (Table 1) but as these trials are still running the results are not yet mature.

**Table 1:** Clinical trials in MPM of checkpoint inhibitors in the surgical setting (Neo-adjuvant and post-surgery)

Trial acronym or title	Trial identifier	Treatment	Phase	Primary objective(s)	Completion date	Report status	References
A Pilot Window-of-opportunity Study of the Anti-PD-1 Antibody Pembrolizumab in Patients With Resectable Malignant Pleural Mesothelioma	NCT02707666	Neoadjuvant pembrolizumab every 21 days for three cycles A PET/CT scan will then assess response to Pembrolizumab followed by surgical resection followed by adjuvant chemotherapy with cisplatin and pemetrexed every 21 days for 4 cycles	I	The primary objective is to assess an increase in interferon-γ, measured via a gene expression profile (GEP), comparing matched pre- and post-treatment samples (IFN-G GEP response), and other correlative analyses will be performed to identify additional candidate biomarkers for benefit or inherent resistance to Pembrolizumab	Estimated Primary Completion Date December 20, 2022 Estimated Study Completion Date December 20, 2025	Interim	[46]
Phase I Trial of Adjuvant Pembrolizumab After Radiation Therapy for Lung-Intact Malignant Pleural Mesothelioma	NCT02959463	Cohort 1: Hemi-thoracic radiation therapy, plus intravenous Pembrolizumab repeated every 3 weeks for up to 2 years in the absence of disease progression or unacceptable toxicity Cohort 2: Palliative radiation therapy over 1–3 weeks followed by intravenous Pembrolizumab similar to cohort 1. After completion of treatment, patients will be followed up at 30 days, every 6 weeks for 48 weeks, then every 12 weeks for up to 5 years	I	To determine the safety and tolerability of pembrolizumab administered after radiation therapy in patients with malignant pleural mesothelioma (MPM) who have not undergone extra-pleural pneumonectomy	Estimated Primary Completion Date May 31, 2020 Estimated Study Completion Date May 31, 2021	Interim	[47]
A Feasibility Trial of Neoadjuvant Cisplatin-Pemetrexed With Atezolizumab in Combination and in Maintenance for Resectable Malignant Pleural Mesothelioma	NCT03228537	Neoadjuvant cisplatin-pemetrexed disodium (pemetrexed)-atezolizumab Surgical resection (extra-pleural pneumonectomy EPP, or pleurectomy with decortication P/D), followed finally by radiotherapy for those patients who underwent EPP. Maintenance atezolizumab will then be given for up to 1 year in the absence of disease progression or unexpected toxicity	I	To evaluate if the regimen of neoadjuvant cisplatin-pemetrexed disodium (pemetrexed)-atezolizumab, surgery ± radiation, then maintenance atezolizumab is feasible and safe for patients with resectable malignant pleural mesothelioma	Estimated Primary Completion Date June 1, 2021 Estimated Study Completion Date June 1, 2021	Interim	[38]
Window Of Opportunity Phase II Study Of MEDI4736 Or MEDI4736 + Tremelimumab In Surgically Resectable Malignant Pleural Mesothelioma	NCT02592551	Durvalumab (anti-PD-L1) or combination therapy with Durvalumab plus tremelimumab (anti-CTLA4) in mesothelioma patients undergoing surgery	II	Primary outcome measures are assessment of the intra-tumoral ratio of CD8 T cells to regulatory T cells (CD8/Treg), the percentage of inducible T-cell co-stimulator (ICOS) + CD4 T cells, and the tumor expression programmed death-ligand 1 (PD-L1)	Estimated Primary Completion Date September 1, 2022 Estimated Study Completion Date September 1, 2022	Interim	[47]
Neoadjuvant Immune Checkpoint Blockade in Resectable Malignant Pleural Mesothelioma	NCT03918252	Cohort 1: Preoperative nivolumab, prior to planned surgery Cohort 2: Preoperative nivolumab + ipilimumab prior to planned surgery	I/II	Safety and Feasibility of neoadjuvant nivolumab ± ipilimumab in patients with resectable MPM	Estimated Primary Completion Date June 2025 Estimated Study Completion Date June 2026		[48, 49]
NICITA: Nivolumab With Chemotherapy in Pleural Mesothelioma After Surgery	NCT04177953	Following cytoreductive surgery (with or without hyperthermic intrathoracic chemoperfusion) patients will be randomized to receive either platinum-based adjuvant chemotherapy (Arm A) or platinum-based adjuvant chemotherapy together with nivolumab (Arm B)	II	The primary endpoint will be time-to-next-treatment (TNT) which has been defined as the time from randomization until the initiation of any additional intervention as a consequence of disease progression, using a Kaplan–Meier analysis	Estimated Primary Completion Date July 2023 Estimated Study Completion Date December 2023	Interim	[50]

### Checkpoint inhibitor immunotherapy in the surgical setting

Aside from the neo-adjuvant setting, there is one ongoing multicenter, randomized, controlled, open-label Phase II study which is designed to assess the efficacy of standard chemotherapy combined with nivolumab in the context of multimodal management of early-stage MPM (Table 1) [50]

### Checkpoint inhibitor immunotherapy within the front-line (first-line) setting

A number of studies have been completed or are ongoing which aim to examine the potential utility of checkpoint inhibitor immunotherapy in the front-line setting. Several of these are ongoing (Table 2), but interim and published results from some of these trials as discussed by us and others [47, 48, 51, 52] suggest that checkpoint inhibitors will be important new agents in the frontline setting for the management of MPM. For example, interim results of PrE0505 (Table 2) demonstrate a median OS of 20.4 months, and a 1-year OS rate of 70.4% [52] with new trials such as DREAM3R (Table 3) initiated on the basis of these interim results.

**Table 2:** Clinical trials in MPM of checkpoint inhibitors within the front-line (first-line) setting

Trial acronym or title	Trial identifier	Treatment	Phase	Primary objective(s)	Completion date	Report status	References
DREAM A phase 2 trial of durvalumab with first line chemotherapy in mesothelioma with a safety run in	ACTRN12616001170415	To investigate the effectiveness of durvalumab in combination with standard chemotherapy for mesothelioma	I/II	PFS at 6 months (PFS6) according to mRECIST for MPM	27/09/2019 (Date of last data collection)	Completed Met its primary endpoint with a 6-month PFS rate of 57% and a median OS of 18.4 months	[51]
PrE0505	NCT02899195	A single arm, open label phase II study of durvalumab, in combination with standard chemotherapy	II	OS assessed in accordance with RECIST 1.1 Time Frame: 72 months	Actual Primary Completion Date: February 16, 2020 Estimated Study Completion Date: June 2023	Interim The median OS at the time of report was 21.1 months. The 12 month OS rate was 70%	[52]
Checkmate-743 A Phase III, Randomized, Open Label Trial of Nivolumab in Combination With Ipilimumab Versus Pemetrexed With Cisplatin or Carboplatin as First Line Therapy in Unresectable Pleural Mesothelioma	NCT02899299	Nivolumab and Ipilimumab vs Pemetrexed and Cisplatin/Carboplatin	III	OS Time Frame: 4.5 years	Actual Primary Completion Date March 25, 2020 Estimated Study Completion Date: April 15, 2022	Interim Significant improvement in OS versus platinum-based standard chemotherapy (HR, 0.74; 95% CI, 0.61–0.89; P = .002). At 22.1 months of follow-up, the median OS was 18.1 months with the combination (95% CI, 16.8- 21.5) versus 14.1 months with chemotherapy (95% CI, 12.5 -16.2). The 2-year OS rates were 41% and 27%, respectively	[53, 54]
DREAM3R DuRvalumab With chEmotherapy as First Line treAtment in Advanced Pleural Mesothelioma	NCT04334759	First-line cisplatin plus pemetrexed with or without durvalumab	III	OS Time Frame: Minimum follow-up is 24 months after randomisation	Estimated Primary Completion Date: April 2025 Estimated Study Completion Date: December 2025	Not yet reported	
A Phase II/III Randomized Study of Pembrolizumab in Patients with Advanced Malignant Pleural Mesothelioma	NCT02784171 (CTG-IND-I227)	Cisplatin/Pemetrexed vs Cisplatin/Pemetrexed + Pembrolizumab vs Pembrolizumab (Phase II only)	II/III	Phase II: PFS measured as time from randomization to first observation of objective disease relapse or progression [Time Frame: 32 months] Phase III: OS defined as time from randomization to the date of death from any cause [Time Frame: 32 months]	Estimated Primary Completion Date: July 31, 2022 Estimated Study Completion Date: December 31, 2022	Not yet reported

**Table 3:** Clinical trials in MPM of single-agent checkpoint inhibitors within the second-line (salvage) setting

Trial acronym or title	Trial identifier	Treatment	Phase	Primary objective (s)	Completion date	Report status	References
MERIT A multi-centre, open-label, uncontrolled, phase II study to investigate efficacy and safety of ONO-4538 in malignant pleural mesothelioma (ONO-4538–41(33–609))	JapicCTI-163247	Nivolumab	II	ORR according to mRECIST	Primary Completion Date: March 14, 2018	Completed ORR: 29% Median OS was 17.3 months	[55, 56]
DETERMINE A Phase 2b, Randomized, Double-blind Study Comparing Tremelimumab to Placebo in Second- or Third-line Treatment of Subjects With Unresectable Pleural or Peritoneal Malignant Mesothelioma	NCT01843374	Temelimumab	II	OS (defined as time from randomisation until death from any cause)	Actual Primary Completion Date: January 24, 2016 Estimated Study Completion Date: December 31, 2020	Completed Median overall survival did not differ significantly between the treatment groups: it was 7·7 months (95% CI 6·8–8·9) in the tremelimumab group vs 7·3 months (5·9–8·7) in the placebo group (HR 0·92, 95% CI 0·76 − 1·12, p = 0·41)	[57]
PROMISE-Meso A Multicentre Randomised Phase III Trial Comparing Pembrolizumab Versus Standard Chemotherapy for Advanced Pre-treated Malignant Pleural Mesothelioma	NCT02991482	Pembrolizumab vs institutional choice single-agent chemotherapy (gemcitabine or vinorelbine) in relapsed MPM patients with progression after/on previous platinum-based chemotherapy, and at progression, patients randomized to chemotherapy were allowed to crossover to Pembrolizumab	III	Progression Free Survival (PFS) Secondary outcome: OS	Estimated Primary Completion Date: December 2020 Estimated Study Completion Date: December 2020	Completed At a median 11.8 month follow-up, median PFS (95% CI) for pembrolizumab was 2.5 compared with 3.4 months for chemotherapy no difference in OS was detected between groups	[58]
NivoMes	NCT02497508	Nivolumab (3 mg/kg) administered every 2 weeks intravenously for a maximum of 12 months or until disease progression or unacceptable toxicity	II	Disease Control Rate (DCR)	Actual Study Completion Date: July 2017	Completed The trial demonstrated a DCR of 50% and an ORR of 24% median OS of 11.8 months	[59]
JAVELIN	NCT01772004	Avelumab at 10 mg/kg every 2 weeks	I	Dose Limiting Toxicity Best OR	Completion Date December 16, 2019 MPM arm completed July 22, 2015	Completed Acceptable safety profile with grade 3 or 4 treatment related adverse event of 9% median OS of 10.7	[60]
Keynote-028 Phase IB Study of Pembrolizumab (MK-3475) in Subjects With Select Advanced Solid Tumors	NCT02054806	Basket trial across 20 different cohorts of patients with PD-L1–positive, advanced solid tumors (including MPM) to assess the antitumor effects of Pembrolizumab given 10 mg/kg every 2 weeks for 2 years or until confirmed disease progression or unacceptable toxicity occurred	I	ORR using Response Evaluation Criteria in Solid Tumors (RECIST Version 1.1) [ Time Frame: Up to 24 months]	Estimated Study Completion Date: December 18, 2023	Interim In 2017 an ORR of 20% was reported in the MPM cohort The same ORR was reported for MPM in 2019 with a median PFS of 5.5 months and a median OS of 18.7 months	[61, 62]
Keynote-158 A Clinical Trial of Pembrolizumab (MK-3475) Evaluating Predictive Biomarkers in Subjects With Advanced Solid Tumors (KEYNOTE 158)	NCT02628067	Experimental arm 1: pembrolizumab 200 mg every 3 weeks (up to 2 years) Experimental arm 2: Patients with high tumour mutational burden (TMB) – excluding those with mismatch repair deficient (dMMR/MSI-H) receive 400 mg every 6 weeks (up to 2 years)	II	Objective Response Rate (ORR) [Time Frame: Up to approximately 2 years]	Estimated Study Completion Date: June 18, 2026	Interim For all cohorts an overall ORR of 29% in TMB high vs 6% in TMB low In MPM an ORR was only observed in 9 of 84 TMB low cases	[63]
IRB14-1381	NCT02399371	Intravenous pembrolizumab every 21 days for up to 24 months in the absence of disease progression or unacceptable toxicity	II	Ability of PD-L1 to predict response Secondary outcomes include: OS, PFS, DCR	Estimated Primary Completion Date:March 20, 2021 Estimated Study Completion Date: March 20, 2023	Interim median OS of 11.5 months	[64]
An Efficacy and Safety Study of Avelumab Plus SBRT in Malignant Mesothelioma (MPM)	NCT03399552	One dose of Avelumab (10 mg/kg) every other week as well as a short course of SBRT after the first two doses of avelumab	I/II	Overall response rate [Time Frame: 3 years] defined by modified RECIST 1.1 for mesothelioma	Estimated Study Completion Date December 2021		

Most recently, analysis of the Checkmate-743 trial (Table 2) has resulted in the FDA approval of a combination therapy of Nivolumab/Ipilimumab as a first line treatment for unresectable MPM [53, 54]. The median OS with this treatment was consistent between patients with epithelioid histology (18·7 months) and non-epithelioid histology (18·1 months) [53]. The OS benefit observed in the non-epithelioid subgroup for the checkpoint inhibitor combination versus standard chemotherapy is notable (18.7 months vs 8.8 months), but can be attributed to the established inferior effect of chemotherapy in the non-epithelioid subtype [53].

The approval of Nivolumab/Ipilimimab by the FDA as a front-line therapy for the treatment of MPM is greatly encouraging [54], and the results of the various ongoing trials will help improve the utility of checkpoint inhibitors in the front-line setting moving forwards.

### Checkpoint inhibitor immunotherapy within the salvage setting

A large number of studies are also currently investigating the potential use of checkpoint inhibitors within the second or third-line (salvage therapy) setting, and are discussed in more detail in the following sections.

#### Single agent checkpoint inhibitor studies

Several clinical trials of checkpoint inhibitors as single agents have been completed in the salvage setting and are summarized in Table 3. In particular, the MERIT trial, a Phase II multi-center, open-label, uncontrolled, trial of patients within the second-line setting, observed an OS of 17.3 months which resulted in Nivolumab being approved by the Japanese Ministry of Health, Labor and Welfare for salvage therapy in MPM [65]. Despite this, several other single-agent trials of checkpoint inhibitors such as DETERMINE, PROMIS-MESO, JAVELIN or Nivo-Mes for example have had mixed results (Table 3) and these data have been reviewed extensively by us and others [19, 47, 66]. Another ongoing single institute phase II trial (IRB14-1381-NCT02399371) has reported interim results suggesting that checkpoint inhibitors show robust activity in the salvage setting, with a median OS of 11.5 months [64] (Table 3). Most recently, interim results from the Phase III trial CONFIRM trial (Table 3) have been presented [67]. In the interim results presented superiority for nivolumab over and above placebo was observed for OS with a hazard ratio of 0.72 (*p* = 0.018). The same was true for PFS with a hazard ratio of 0.61 (*p* < 0.001). Interestingly PD-L1 expression had no bearing on OS, whereas an epithelioid histology was found to have a significant survival advantage with a 12 month OS (40 vs. 26.7 months) with a hazard ratio of 0.71 (*p* = 0.021) [67]. Despite this, data from the Dutch expanded access program, suggest that in a real-world setting patients with recurrent malignant pleural mesothelioma, nivolumab did not provide the same benefits as observed in clinical trials with worse ORR and a median OS of only 6.7 months [68, 69].

#### Combination checkpoint inhibitor studies in MPM

Several studies have now combined checkpoint inhibitors in the salvage setting summarized in Table 4.

**Table 4:** Clinical trials in MPM of combined checkpoint inhibitors within the second-line (salvage) setting

Trial acronym or title	Trial identifier	Treatment	Phase	Primary objective(s)	Completion date	Report status	References
MAPS2	NCT02716272	Monotherapy vs Combination Therapy Nivolumab 3 mg/kg every 2 weeks vs Nivolumab as above plus Ipilimumab 1 mg/Kg every 6 weeks	II	DCR assessed by CT scan [Time Frame: 3-months] Tumor assessment (modified RECIST1.0 for mesothelioma) Secondary outcomes included OS, PFS	Estimated Study Completion Date: April 2020	Completed Median OS data observed was 11.9 months for the single therapy and 15.9 months for the combination	[70]
NIBIT-Meso-1	NCT02588131	Single arm Tremelimumab 1 mg/kg plus Durvalumab 20 mg/kg every four weeks for 4 doses, then Durvalumab 20 mg/kg every four weeks for an additional 9 doses	II	immune related ORR Secondary objectives included OS, PFS and DCR	Estimated Study Completion Date: March 2018	Completed median OS for the study was 16·6 months	[71]
INITIATE	NCT03048474	Nivolumab (240 mg flat dose every 2 weeks) plus Ipilimumab (1 mg/kg every 6 weeks up to four times)	II	Disease Control Rate (DCR) at 12 weeks Secondary Outcomes included PFS, OS, ORR	Actual Study Completion Date: December 2019	Completed Median overall survival was not reached (estimated to exceed 12·7 months)	[72–74]
A Phase 2 Study of Durvalumab in Combination With Tremelimumab in Malignant Pleural Mesothelioma	NCT03075527	Single arm Durvalumab plus Tremelimumab once per day for every 28 day cycles (+ 7 days). Participants will receive tremelimumab for up to 4 cycles (4 doses). Beginning with cycle 5 day 1, subjects will continue to receive durvalumab alone, until clinical or radiological progression	II		Actual Primary Completion Date: June 7, 2019 Estimated Study Completion Date: September 30, 2024	Suspended At a median follow-up of 7.1 months, the median PFS was 2.8 months, and the median OS was 7.8 months	[75]

Two of these trials (MAPS2, NIBIT-Meso-1—Table 4) documented responses with a median OS of approximately 16 months for the combination arms [70, 71].

The INITIATE trial (Table 4) which had an estimated OS of approximately 12.7 months (Table 4) [72–74] along with the NivoMes trial (Table 3) were recently re-examined to complete a comprehensive immune cell profiling of samples [74], and the results demonstrated that the combination therapy induced a profound increase in the proliferation and activation of effector memory T cells which was not observed in the monotherapy, suggesting a clear benefit for the combination therapy, and therefore this observation warrants a larger Phase III trials of this combination therapy in the salvage setting.

Other trials of combination therapies in the combination setting have not shown as good responses. For example NCT03075527 a single institute trial examining a Durvalumab/ Tremelimumab combination was prematurely terminated as it did not meet its primary endpoint of ORR at interim analysis [75] (Table 4). The results of these trials continue to support the further development of checkpoint inhibitors as both single agents or as combination therapies in MPM.

### Are there other checkpoint inhibitor therapy options?

However, checkpoint inhibitors and anti-tumor immunity are not restricted to just the three candidates (CTLA-4, PD-1, PD-L1) currently described in the previous sections. Many other potential immunotherapy targets have been identified as shown in Table 5, and some potentially actionable candidates are discussed in the following sections.

**Table 5:** Known checkpoints examined for expression and survival in MPM

Gene	Alternative names	Altered expression^a^ (mRNA)	OS^b^ (mRNA)	Comments
CD244	2B4	No	No	
TNFRSF9	4-1BB	No	No	
ANGPTL7	CDT6	No	No	
CD80	B7-1	No	No	
CD86	B7-2	No	No	
ICOSLG	B7-H2	No	No	
**CD276**	**B7-H3**	**N/A**	**Yes** **(p = 0.039)**	**High expression associated with poor OS**
VTCN1	B7-H4	No	No	
HHLA2	B7-H5	No	No	
NCR3LG1	B7-H6	N/A		Not found in UALCAN
BTLA		N/A	No	
BTN1A1	BTN	No	No	
**BTN3A1**	**CD277**	**Yes** **(p = 0.029)**	**Yes** **(p = 0.0093)**	**Upregulated in MPM** **High expression associated with better OS**
**BTN3A3**	**BTF3**	**Yes** **(p = 0.042)**	**Yes** **(p = 0.0065)**	**Upregulated in MPM** **High expression associated with better OS**
**PVR**	**CD155**	No	**Yes** **(p = 0.023)**	**High expression associated with poor OS**
CD160	BY55	No	No	
LY9	CD229	No	No	
CD28	Tp44	No	No	
TNFRSF8	CD30	No	No	
CD40	TNFRSF5	No	No	
**CD47**	**MER6, IAP**	**Yes** **(p = 0.012)**	No	**Upregulated in MPM**
CD48	BLAST1	No	No	
**CD84**	**SLAMF5**	No	**Yes** **(p = 0.042)**	**High expression associated with poor OS**
CD96	TACTILE	No	No	
CTLA-4	CD152	No	No	
CD226	DNAM-1	No	No	
LGALS9	Galectin-9	No	No	
TNFRSF18	GITR	N/A	No	
**TNFRSF14**	**HVEM**	No	**Yes** **(p = 0.019)**	**High expression associated with better OS**
TIM3	HAVCR2	No	No	
ICOS	AILIM	No	No	
**LAG3**	**CD223**	**Yes** **(p = 0.011)**	**Yes** **(p = 0.021)**	**Upregulated in MPM** **High expression associated with better OS**
LAIR-1		No	No	
LAIR-2	CD306	No	No	
**LILRA2**	**LIR7, CD85H**	No	**Yes** **(p = 0.038)**	**High expression associated with better OS**
LILRA3	LIR4, CD85E	No	No	
LILRA5	LIR9, CD85F	No	No	
LILRB1	LIR1, CD85	No	No	
LILRB2	LIR2, CD85D	No	No	
LILRB3	LIR3, CD85A	No	No	
LILRB4	LIR5, CD85K	No	No	
LILRB5	LIR8, CD85C	No	No	
**Nectin-1**	**PVRL1**	No	**Yes** **(p = 0.0042)**	**High expression associated with poor OS**
Nectin-2	PVRL2	No	No	
**Nectin-3**	**PVRL3**	**Yes** **(p = 0.012)**	**Yes** **(p = 0.024)**	**Upregulated in MPM** **High expression associated with poor OS**
NCR3	NKp30	No	No	
SLAMF6	NTB-A	N/A	No	
OX40	TNFRSF4	No	No	
**OX40L**	**TNFSF4**	**Yes** **(p = 0.047)**	**Yes** **(p = 0.00018)**	**High expression associated with poor OS**
PD1	CD279	No	No	
PD-L1	CD274	N/A	No	
PD-L2	B7DC	No	No	
PVRIG	CD112R	No	No	
**SIRPA**	**PTPNS1**	No	**Yes** **(p = 0.0091)**	**High expression associated with better OS**
SIRPG	SIRP gamma	No	No	
**SIRPB1**	**SIRP-beta 1**	**Yes** **(p = 7.92 × 10**^**–7**^**)**	No	**Upregulated in MPM**
SIRPB2		N/A	No	
SLAMF1	CD150	No	No	
SLAMF7	CD319	No	No	
TIGIT		N/A	No	
**VISTA**	**VSIR, C10ORF54**	**N/A**	**Yes** **(p = 0.00093) **	**High expression associated with better OS**
VSIG3	IGSF11	N/A	No	Ligand of VISTA
VSIG4	CRIG	No	No	

Bold value represents significance at p < 0.05

^a^Assessed using oncomine analysis [76] of the Gordon MPM dataset (normal pleura versus malignant) [77]

^b^Assessed using UALCAN [78]

**LAG-3:** Lymphocyte activation gene-3 (LAG-3, also known as CD223) is a checkpoint inhibitor, where it acts as an inhibitory co-receptor, playing pivotal roles in autoimmunity, tumor immunity, and anti-infection immunity [79]. A number of agents targeting this receptor are in active clinical development [79]. LAG-3 has been proposed as a candidate checkpoint inhibitor target in MPM [80], and expression of LAG-3 has been identified on immune cell infiltrates isolated from patients with MPM [81]. Most recently a study found that whilst the immune phenotype of pleural fluid cells had no prognostic significance, the presence of PD-1 + /LAG-3 + /TIM-3 + CD4 + tumor-infiltrating lymphocytes in pleural biopsy samples correlated with worse overall survival [82]. Intriguingly, analysis of The Cancer Genome Atlas (TCGA) dataset indicates that high mRNA expression of LAG-3 is associated with better OS (Table 5), and a recent analysis suggests that high LAG-3 mRNA expression is a common feature in mesothelioma at both the mRNA and protein level [83, 84].

In a recent report Marcq et al*.*, have shown that in pre-clinical models of MPM, a combination of an anti-PD-1/anti-LAG-3 results in delayed tumor growth and survival benefit [85]. Interestingly, a bispecific antibody Tebotelimab (in development by Macrogenics) targets both LAG-3 and PD-1 and is currently in a Phase I dose escalation study (NCT03219268). Preliminary data from this study suggests it has an acceptable safety profile with encouraging early evidence of anti-tumor activity, with one confirmed partial response in a mesothelioma patient [86].

**VISTA:** V-type immunoglobulin domain-containing suppressor of T cell activation (VISTA) (also known as VSIR or B7H5) is an immune-checkpoint gene which was first reported as having strong expression in epithelioid MPM, above and beyond that seen in other solid cancers, with obvious implications for the immune response to MPM and for its immunotherapy [87]. A subsequent study has confirmed that VISTA expression is higher in epithelioid subtype [88, 89]. High expression of VISTA in epithelioid cancers is associated with a better OS, in both the TCGA dataset (Table 1) and in a separate analysis in the French MESOBANK samples [90]. CA-170 is a small molecule inhibitor of VISTA in development by Curis, and NCT04475523 is an open-label, multicenter dose-escalation study of CA-170, assessing the safety and tolerability of this agent in patients with relapsed/refractory solid tumors. This trial had a cohort of (n = 12) MPM patients, and the results for this cohort were recently presented, which effectively showed that while CA-170 was well tolerated and showed favorable clinical pharmacokinetics, no partial or complete responses were reported in MPM [91].

**B7-H3:** B7H3 (also known as CD276) is another candidate checkpoint, whose expression has been observed in mesothelioma [92]. In 2018, it was reported that expression of B7H3 was positive in 41 of 44 mesothelioma samples tested, and of these 39/44 highly expressed B7H3 [93]. The histological subtype of the mesothelioma specimens examined was not provided. A separate study has confirmed that almost all MPM patients across all histological subtypes were positive for B7-H3 (epithelioid − 90.9%; non-epithelioid − 88.9%) [94]. In this analysis albeit of a small number of patients (n = 31), it was found that the expression level of B7‐H3 was significantly higher than that of PD‐L1 in the epithelioid type, whereas in non‐epithelioid samples, there was no significant difference in the expression levels of PD‐L1 and B7‐H3 [94]. Analysis of the TCGA dataset demonstrates that high expression of B7H3 mRNA is associated with a worse OS (Table 1).

Several compounds targeting B7-H3 are under active development by companies such as Daichii-Sankyo (DS-7300—a humanized antibody drug (topoisomerase inhibitor) conjugate) or Macrogenics (Enoblituzumab/MGA271—monoclonal antibody; MGC018—a humanized monoclonal antibody (DNA alkylating agent) conjugate). All are currently in Phase I/II clinical trials. Interim data from the MGC018 trial (NCT03729596) has been presented which indicate that this antibody drug conjugate (ADC) has a manageable safety profile with early evidence of clinical activity [95].

**TIM3:** The T-cell inhibitory receptor Tim3 (T-cell immunoglobulin and mucin-domain containing-3) is a heavily investigated immune- checkpoint [96], and demonstrating significant pre-clinical activity [97, 98]. Tim3 expression has been examined in MPM, and its expression is found on both tumor cells and immune cells [81, 99], and double-positive PD-1 + /TIM-3 + CD8 + T cells are more commonly found in PD-L1–positive tumors [99]. Whilst expression of this receptor does not have any prognostic value (Table 1) in the MPM TCGA dataset, its expression suggests that it may be a potential new target in mesothelioma [85, 100].

**TIGIT:** The role of inhibitory repressors (IRs) on tumor infiltrating lymphocytes (TILs) is generally associated with T-cell exhaustion. In such a situation, when exposed to chronic tumor antigens, T cells become dysfunctional/exhausted and upregulate various checkpoint inhibitory receptors (IRs) that limit their survival and function [101]. In a recent analysis of TILs isolated from patients with MPM [102], it was observed that the levels of TIGIT were significantly greater on TILs isolated from MPM compared with those isolated from tumor free lungs (TFLs), with high levels of TIGIT on ~ 60% of CD8 + T-cells [102]. Functionally, the expression of TIGIT was associated with TIL hypofunction [102], suggesting that an anti-TIGIT therapy may have potential for therapeutic use in mesothelioma [102], and a number of clinical trials and anti-TIGIT therapies are in progress [103].

**BTN3A1/ BTN3A3**: Butyrophilin subfamily 3 (BTN3) genes are emerging as checkpoints critical to the regulation of immune responses for specific γδ T cell (Vγ9Vδ2T) subsets which can exert anti-tumoral effects [104]. Two of these BTN3A1 (CD277) and BTN3A3 (BTF3) are upregulated in MPM and high expression is associated with MPM OS (Table 5). Vγ9Vδ2T cell infiltration into tumor tissues is associated with a positive prognosis across multiple cancers [105], which makes the BTN3A subfamily an interesting target for enhancing anti-tumor immunity. Several companies have developed agents targeting butyrophilins. One candidate is a humanized anti-Butyrophilin 3A (BTN3A) monoclonal antibody (ICT01) developed by ImCheck Therapeutics and which is currently in a Phase I/IIA (NCT04243499) first-in-human, open-label clinical trial to characterize the safety, tolerability and activity of as monotherapy and in combination with Pembrolizumab in patients with advanced, relapsed/refractory cancer, including both solid tumors and hematologic cancers. Preliminary data from the first dose cohort of patients with solid tumors were recently presented and show a favorable safety profile with robust activation and migration of γ9δ2 T cells at doses as low as 1 μg/kg [106].

**OX40/OX40L:** These are members of the TNF receptor superfamily (TNFRSF), and are key co-stimulators of T cells during infection, and there has been an increasing interest in harnessing these receptors to augment tumor immunity. OX40 (TNFRSF4) and OX40L (TNFSF4) have been implicated in mesothelioma. In a recent study of an animal model of mesothelioma, tumor resident regulatory T-cells were shown to co-express high levels of CTLA-4 and OX40 on a large proportion of cells. Individually targeting OX40 generated an effective response against tumor development, and was found to be synergistic with anti-CTLA4 agents [107]. Whilst there appears to be little information as regards OX40L in mesothelioma, analysis suggests that OX40L is overexpressed in MPM and high expression is associated with poorer OS (Table 1). At present a Phase I clinical trial (NCT03894618) of SL-279252 (PD1-Fc-OX40L) is assessing the safety, tolerability, pharmacokinetics, anti-tumor activity and pharmacodynamic effects of this bi-functional fusion protein [108] in patients with advanced solid tumors or lymphomas. The trial is expected to complete in April, 2022.

Other candidate checkpoints which could be therapeutically targeted include PVR (CD155), CD47 (MER6, IAP), CD84 (SLAMF5), TNFRSF14 (HVEM), and various members of the nectins (Table 5). Clearly, as our knowledge of checkpoint inhibitor therapy improves, the wealth of candidate targets and agents currently under investigation coupled with emerging data from patients with MPM suggest that further investigations of combination immune checkpoint inhibitor therapy are warranted.

## Beyond checkpoint inhibitors

### Oncolytic therapy?

While early studies of interferon or GM-CSF based MPM therapy based on infusions proved disappointing, new therapeutic strategies which involve oncolytic virus mediated expression of these agents may have more clinical activity and benefit.

**Oncolytic adenovirus overexpression of IFN**: Several Phase I trials involving intra-pleural infusion of adenoviral mediated interferon therapy have been attempted in recent years [109–111]. In the most recent of these (ClinicalTrials.gov NCT01119664), 40 patients received two intra-pleural doses of a replication-defective adenoviral vector containing the human IFNα2b gene (Ad.IFN) concomitant with a 14-day course of celecoxib followed by chemotherapy (either first line with pemetrexed, or second-line with pemetrexed or gemcitabine. whilst patients in the first-line cohort had median OS of 12.5 months, in second-line settings the median OS was 21.5 months with 32% of patients alive after 2 years [109]. A new Phase III study—(INFINITE—NCT03710876) is currently recruiting for a trial involving intra-pleural administration of TR002 an adenovirus-delivered Interferon Alpha-2b (rAd-IFN) and examining its efficacy and safety in combination with celecoxib and gemcitabine in patients with mesothelioma.

**Oncolytic measles virus overexpression of IFN**: On a separate note, in 2015 defects within the interferon type-I response were found to render MPM cells sensitive to oncolytic measles virus [112], and a follow up study found that the defects in IFN-I responses that renders them sensitive to oncolytic activity induced by exposure to the measles virus were most frequently homozygous deletions of all the 14 IFN-I genes (IFN-α and IFN-β) [113]. These results suggest that the interferon pathway continues to be potentially important immunotherapy target in MPM.

Intriguingly, a recent report indicates that IFN-γ treatment of mesothelioma cells results in both the upregulation of membranous PD-L1 [114], which suggest that interferon therapy, could be combined with anti-PDL1 checkpoint inhibitors for the treatment of MPM.

**Oncolytic adenovirus overexpression of GM-CSF**: ONCOS-102 is an immune-priming GM-CSF coding oncolytic adenovirus in development by Targovax. The safety, immune and clinical results of an open-label Phase I/II clinical trial of ONCOS-102 in combination with pemetrexed/cisplatin (NCT02879669) for 1st and 2nd line unresectable MPM have just been reported, and indicate that the immune priming function of ONCOS-102 was both safe and had robust immune activation, with increased T-cell infiltration [115]. Moreover up-regulation of PD-L1 was noted, which could potentially allow for future combinations with checkpoint inhibitors [115]. Currently, Targovax has been granted a European Patent for combining this oncolytic virus with checkpoint inhibitors (European Patent no 3293201) [116], and has further announced a collaboration with Merck to evaluate ONCOS-102 with Pembrolizumab in MPM [117]. The envisaged trial will be a randomized phase II of up to 100 patients comparing this investigational triple combination against Pembrolizumab and SOC, with multiple centers in both the USA and EU participating, and the aim will be to start enrolling patients into the trial within twelve months. Moving forwards it will be interesting to see the results of any clinical trials combining ONCOS-102 and checkpoint inhibitors.

### CAR-T based approaches

Chimeric antigen receptors (CARs) therapy functions by coupling the Human leukocyte antigen (HLA)-independent binding of a cell surface target to the delivery of a tailored T-cell activating signal by recognizing and binding to specific tumor-associated antigens [118, 119]. The potential to use CAR-T therapy in mesothelioma has been explored fairly extensively, and pre-clinical models using various targets including mesothelin (MSLN) [120, 121], Fibroblast activation protein (FAP) [122], Met Proto-oncogene (cMET) [123], pan-ErbB [124] and others have been extensively tested [125, 126].

Various clinical trials of CAR-T based approaches in MPM have been conducted and were recently summarized by us and others [47, 118, 119, 127].

One factor which may currently limit the use of CAR-T strategies in solid tumors could be the issue of T-Cell exhaustion [128]. However, recent studies suggest that checkpoint inhibitors may be a mechanism for improving the potency of CAR-T cell therapies in this regard [129–131], and other approaches such as co-stimulation induction and cytokine based approaches may also have merit [128].

Dendritic cell (DC) therapy is a cell based vaccination approach used to initiate an anti-tumor immune response [127]. In mesothelioma initial approaches used autologous tumor lysate loaded DCs, and have showed excellent long lasting clinical responses with survival up to 66 months post treatment [132–136]. While greatly encouraging, the main disadvantage of this approach remains that it is time-consuming and may not often generate sufficient amounts of the required quality for DC therapy. Allogenic tumor lysates have the possibility to circumvent this drawback [137], and a Phase I clinical trial MesoCancerVa (NCT02395679) has recently completed. In this trial, no dose-limiting toxicities were established and radiographic responses were observed. The median PFS was 8.8 months and median OS was not reached at a median follow-up of 22.8 months [137]. In a follow up analysis of the peripheral blood T cell receptor β (TCRβ) chain repertoire of nine MPM patients before and 5 weeks after the start of dendritic cell (DC)-based immunotherapy, it was found that clinical responses to DC-mediated immunotherapy was dependent on both the pre-existing TCRβ repertoire of total CD3 + T cells and on therapy-induced changes, in particular expanding PD1 + CD8 + T cell clones, and therefore TCRβ repertoire profiling could potentially allow for the selection of MPM patients that might benefit from DC-based immunotherapy [138].

These promising results have led to the establishment of the Phase II/III DENIM trial (NCT03610360) which aims to recruit n = 230 patients to examine the OS in patients treated with DCs loaded with this allogeneic tumor cell lysate as maintenance treatment after chemotherapy [139]. This trial is estimated to complete in January, 2021, and the results will be eagerly awaited.

## Outstanding issues and other therapeutic considerations

Clearly immunotherapy will in the future play important roles in the management of this cancer. As we continue to develop our understanding and knowledge of these exciting therapeutic options and avenues of approach, additional issues and possibilities arise summarized in Table 6, and are discussed in more detail in the following sections.

**Table 6:** Additional areas of interest within mesothelioma immunotherapy

Outstanding areas of interest for immunotherapy in MPM	
1	Can we combine Tumor-Treating Fields (TTF) with checkpoint inhibitors?
2	How can we best stratify patients to checkpoint inhibitors?
3	Is there any utility for the use of PD-L1 expression as a biomarker to direct therapy?
4	Can we epigenetically prime MPM for checkpoint inhibitor therapy?
5	Can we use BAP1 status in immunotherapy of MPM?
6	Would targeting Toll Like Receptors (TLRs) along with checkpoint inhibitors prove beneficial?
7	What is the best way to monitor response to checkpoint inhibitors?
8	Is the cost prohibitive for the use of checkpoint inhibitors in the second-line/salvage setting?

### Tumor-treating fields and checkpoint inhibitors

Developed by Novocure, the NovoTTF™-100L System is a device which uses alternating electric fields at specific frequencies and intensities (called Tumor Treating Fields or TTF) to selectively disrupt mitosis in cancerous cells [140]. This technology has received FDA approval for use in MPM [141], though concerns exist as to whether potential inherent biases and lack of sufficient controls can allow for a true interpretation of the therapeutic value of this system in MPM [141–144].

Novocure has recently initiated Phase III clinical trials (e.g. NCT02973789) of its platform in combination with immune checkpoint inhibitors in NSCLC [145]. If these clinical trials show efficacy it will interesting to see if similar clinical trials of the NovoTTF™-100L System combined with checkpoint inhibitors will be conducted with MPM moving forwards.

### Patient stratification: Is there a role for tumor mutational burden in predicting response to immunotherapy?

Tumor mutational burden (TMB) is emerging as a strong predictor for identifying cohorts of patients who may respond to checkpoint inhibitor based therapy [146]. Theoretically, a higher TMB should therefore increase the likelihood for tumor neo-antigen production and as such the probability for immune recognition and tumor cell killing [147]. Even though MPM is considered to have a low TMB [48, 148, 149], TMB has been assessed is some available studies of checkpoint inhibitors.

Keynote-028—Expanding on a more detailed analysis of the entire trial cohort (n = 475) it was found that T-cell-inflamed gene expression profiles (GEP), PD-L1 expression and/or tumor mutational burden was associated with a higher likelihood of response to therapy. Within this analysis of the n = 25 mesothelioma patients n = 19 had GEP; n = 12 had PD-L1 positivity and n = 9 had TMB data available [61]. However, no subgroup analysis was available for the mesothelioma cohort alone.

In an analysis of the Keynote-158 study with a pre-specified cutpoint of at least 10 mutations per megabase as TMB-high, 9/84 MPM patients who were assessed as being TMB-low had an ORR [63], although in terms of PFS and OS TMB-high status with Pembrolizumab treatment was not significant for the overall population [150]. In a single case study, an MPM patient who derived a prolonged response to a checkpoint inhibitor (45 months to 52 cycles of Pembrolizumab) was also assessed for TMB. The baseline biopsy was found to have 0.92 somatic mutations per megabase, while the relapse biopsy had 0.26 [151]. The issue of TMB therefore remains to be resolved for its potential utility in predicting or stratifying MPM patients to checkpoint inhibitor based immunotherapy.

### Is there any utility for the use of PD-L1 expression as a biomarker to predict response?

The role of PD-L1 expression as a biomarker to predict outcome in MPM is still an ongoing issue that has yet to be resolved. If one considers the results of Checkmate-743, PD-L1 status does not predict for OS benefit as similar survival was seen in the subgroups with less than 1% vs 1% or greater PD-L1 status [53]. Similar results have been observed in other clinical trials [58, 59, 71]. The results of the MERIT trial found that differences in OS and PFS favored positive PDL-1 expression (although not-significant) [55]. What is emerging from these studies is that expression of PD-L1 is associated with higher ORR [60, 69, 70, 72, 152], and in analysis of the Dutch expanded access program, long survival for patients with partial responses suggested a clinical benefit that is correlated with ORR [69]. Moreover, expression of PD-L1 and non-epithelioid histology is associated with higher ORR [55, 69]

One feature that emerged from Checkmate-743 was that patients who had tumor PD-L1 expression of less than 1% had better survival with chemotherapy which suggests that absence of PD-L1 might be indicative for chemotherapy based regimens. Support for this comes from a recent analysis of the immune microenvironment in MPM which identified that chemotherapy treated patients deriving the best OS were PD-L1 negative and had a higher percentage of stromal CD8 + lymphocytes [153, 154]. Likewise, the Dutch nivolumab EAP study also found that patients no PD-L1 expression had very poor responses to Nivolumab with significantly worse ORR and mOS [68, 69].

Other interesting developments as regards PD-L1 expression as a candidate biomarker are emerging from the CONFIRM trial which found that PD-L1 expression had no bearing on OS [67].

As such PD-L1 remains a contentious biomarker in this sphere, and a significant number of patients exist who whilst being PD-L1 negative, demonstrate ORRs to checkpoint inhibitors. The challenge will be to identify new markers or ways to identify such patients, perhaps using transcriptomic or other approaches [53, 154–157].

### Is there a role for epigenetic priming in the use of checkpoint inhibitor therapy for MPM?

Epigenetic priming is emerging as a mechanism to potentially prime solid tumors for enhanced targeting of immune checkpoint inhibitors via the induction or upregulation of PD-L1. It is now well established that epigenetic targeting agents such as decitabine (a DNA methyltransferase inhibitor) can induce or upregulate PD-L1 expression [158, 159]. In this regard a clinical trial (NCT03233724) designed primarily for NSCLC (but includes MPM) (Table 7), aims to assess if epigenetic targeting with Decitabine can prime solid tumors for enhanced targeting of immune checkpoint inhibitors (in this instance Pembrolizumab) [160].

**Table 7:** Additional clinical trials in MPM utilizing checkpoint inhibitors

Trial acronym or title	Trial identifier	Treatment	Phase	Primary objective (s)	Completion date	Report status	References
Phase I/II Evaluation of Oral Decitabine/Tetrahydrouridine as Epigenetic Priming for Pembrolizumab Immune Checkpoint Blockade in Inoperable Locally Advanced or Metastatic Non-Small Cell Lung Cancers, and Esophageal Carcinomas, or Pleural Mesotheliomas	NCT03233724	Experimental: 1 (Dose Escalation) Decitabine (DAC) –Tetrahydrouridine (THU) + pembrolizumab at escalating doses Experimental: 2 Dose Expansion DAC-THU + pembrolizumab at the dose established in Arm 1	I/II	Maximum Tolerated Dose (MTD) ORR – to determine if the combination is associated with an ORR which exceeds that of Pembrolizumab alone in patients who have PD-L1 expression of at least 50% and those who do not	Estimated Study Completion Date: December 31, 2026	Running no interim results as yet	
ORIGIN Overcoming Resistance to Immunotherapy Combining Gemcitabine With Atezolizumab in Advanced NSCLC and Mesothelioma Progressing Under Immune-checkpoint Inhibitors or Gemcitabine. A Multicenter, Single-arm, Open Label Phase II Trial With Two Cohorts	NCT04480372	Cohort 1. NSCLC Cohort 2. Inoperable MPM gemcitabine 1000 mg/m2 on day 1 and day 8 of each cycle (every 3 weeks) and with atezolizumab 1200 mg on day 1 of each cycle (every 3 weeks)	II	ORR	Estimated Primary Completion Date: April 2025 Estimated Study Completion Date: December 2025	Not yet recruiting	

Other epigenetic targeting agents such as histone deacetylase inhibitors (HDACi) are also well established as candidate agents with the capacity to induce PD-L1 in cancer cells [161–164]. However, in MPM cell lines HDACi by themselves had modest effects on PD-L1, but when combined with decitabine, higher induction of this checkpoint inhibitor were observed [165].

### Can a patients BAP1 status inform therapy decisions?

Given the potential sensitivity of BAP1 mutated MPM to Enhancer of Zeste 2 (EZH2) inhibitors [166], is there an opportunity to combine EZH2 inhibitors with checkpoint inhibitors? In a non-mesothelioma setting, a patient with SMARCB1-deleted, metastatic, poorly differentiated chordoma was treated with Tazemetostat (EZH2 inhibitor), and had a significant increase in intratumoral and stromal infiltration by immune cells expressing checkpoint regulators PD-1 and LAG-3 [167]. In this regard, preliminary data from the EZH-203 (NCT02860286) trial of Tazemetostat in MPM had a 12 week DCR of 47% (n = 35), with mostly stable disease with no complete responses and only 2 partial responses [168]. Given the observation that Tazemetostat results in enhanced infiltration of immune cells it may be possible to conceive of a strategy which could include Tazemetostat/anti-PD1 in BAP1 mutant patients. In this regard, a recent study has shown that while macrophages can be directly cytotoxic for mesothelioma cells, inhibition of EZH2 reduced that activity because it induced PD-1 overexpression. A combination of PD-1 blockade and EZH2 inhibition restores macrophage cytotoxicity [169]; and suggests that combination therapy with EZH2 inhibitors plus checkpoint inhibitors may have potential for clinical efficacy in MPM.

For those patients with wild-type BAP1, there may be a possibility to combine gemcitabine with immunotherapy. Initial pre-clinical studies suggest that it did not change the expression of PD-L1 on human mesothelioma cell lines in vitro [170]. Additional evidence now suggests that wild-type (WT) BAP1 positivity may be a factor in the sensitivity of MPM to gemcitabine [171, 172]. Moreover, a recent study using PET demonstrated that gemcitabine based therapy in a murine colon cancer model strongly induced PD-L1 Expression [173]. Furthermore, a synergistic effect for gemcitabine combined with anti-PD1 was observed in pre-clinical models of mesothelioma, and similar responses were seen in two patients who were resistant to gemcitabine or anti-PD-1 (Pembrolizumab) as monotherapy, but who achieved observable clinical responses following combination therapy [170], and has led to the ORIGIN trial (Table 7), which will examine if a Gemcitabine/Atezolizumab combination can overcome resistance in either advanced NSCLC or Mesothelioma patients progressing under immune-checkpoint inhibitors or gemcitabine. Intriguingly, gemcitabine has been shown by us to act as a DNA methyltransferase inhibitor, reactivating silenced genes in mesothelioma cells [174], and as such the observed responses to gemcitabine on PD-L1 expression changes may reflect an epigenetic priming event, although functional studies will be required to delineate this.

### Can combined targeting of TLRs and checkpoint inhibitors improve responses to immunotherapy?

Toll-like receptors (TLRs) are expressed on many innate immune system cells and play a role in maturation of dendritic cells and priming of cytotoxic T lymphocytes [175]. A subset of TLRs has been shown to stimulate antitumor responses, and agonists to these receptors are being investigated in clinical trials [175, 176]. Several studies have linked TLR3, TLR7 and TLR9 as potentially targetable in MPM [177–179], which could conceivably be trialed in combination with checkpoint inhibitors [180]. It is interesting to note that NCT02668770 is a clinical trial of ipilimumab and MGN1703 (a TLR Agonist) currently running in patients with advanced solid malignancies. Whether any mesothelioma patients are in this trial is unknown.

### What is the best way to monitor immunotherapy response?

Hyper-progression, an accelerated growth or progression of a cancer after treatment is initiated, has been observed for a subset of patients undergoing checkpoint inhibitor therapy [181], and can emerge either during therapy, or can emerge post-therapy [182, 183]. This further complicated by the issue of pseudo-progression where patients obtain an objective response following an initial progression with immunotherapy [184] The estimated occurrence of hyper-progression is estimated at 4 to 29%, while that of pseudo-progression ranges from 0 to 15% [184]. Whilst there is little evidence that hyper-progression occurs during treatments of mesothelioma, two patients have reported as showing pseudo-progression under treatment with Pembrolizumab, within the first 15–30 weeks of therapy followed by responses [185]. Additionally in the DREAM trial, two patients (4%) were also observed to undergo pseudo-progression in response to treatment with Durvalumab [51].

In a recent editorial on this topic key issues remain such as: why it occurs; is it simply a lead‐time bias phenomenon; does it have a strong biological basis such as clonal selection; can we identify and predict those in whom it will occur; and if be stopped by additional therapies [186]. As more and more clinical trials of immunotherapies complete in mesothelioma, vigilance will be required to assess if hyper-progression does occur in MPM while undergoing treatment with immunotherapy.

Some efforts have been made to differentiate pseudo-progression from progression and hyper-progression, such as radiological responses DNA [184]. As PET/CT imaging has been used for the prediction of survival in response to Pembrolizumab in mesothelioma [187], and may be useful to incorporate into immunotherapy based regimens for the treatment of mesothelioma. Other methods that have been explored in other cancer types have involved analyses circulating-tumor DNA or cell-free DNA to assess response to immunotherapy, but larger prospective cohort studies will be required to confirm their potential use [184]. Pathologic scoring of responses to immunotherapy has also been explored [188, 189], but may have limited utility in distinguishing between pseudo-, hyper- and progression in MPM. Clearly, new methods or modalities to monitor immunotherapy response will be required moving forwards.

### Is the cost prohibitive for the use of checkpoint inhibitors in the second-line/salvage setting?

The combined cost for Ipilimumab/Nivolumab in the USA has been estimated approximately $153,800 for four cycles, while that of Nivolumab alone would be of the order of $87,000 [190]. One of the most commonly used chemotherapies in the treatment of MPM in the second-line or salvage setting is vinorelbine [191], which has been estimated to cost $515 for 24 weeks [190]. Given that the recent Dutch EAP program for Nivolumab in pre-treated MPM patients demonstrates a median OS of 6.7 months [68, 69], whilst most trials of vinorelbine in the same setting have a median OS of approximately 9–11 months [191, 192], the question arises if the cost of checkpoint inhibitors in the second line setting will limit use.

## Conclusions

The following sections have described the current state-of-play as regards immunotherapy in MPM. A significant number of studies are investigating checkpoint inhibitors as both monotherapy or in combination therapy in both the front-line and salvage settings. Treatment combinations designed to recruit more immune cells to the tumor such as oncolytic viruses or those that target the interferon pathway hold promise. CAR-T therapy is emerging as a new avenue of approach for immunotherapy in MPM.

Despite impressive durable responses, immune checkpoint inhibitors do not provide a long-term benefit to the majority of patients with cancer [193]. The data arising from immune checkpoint inhibitor studies in MPM has resulted in one FDA approval for a combination checkpoint inhibitor for the first-line treatment of unresectable MPM. The only other approval is for second-line therapy in the salvage setting and is restricted to Japan. Overall, this would suggest that these agents will shortly become part of the frontline treatment options for MPM in the coming years. Given the data from Checkpoint-743 it would seem that nivolumab/ipilimumab should be used in the first line setting, however, cost reimbursement may limit their uptake [194]. The issue of whether or not to give it to all comers irrespective of histology and PD-L1 status has however yet to be resolved given the data that suggests PD-L1 negative tumors have better responses to chemotherapy, and that patients with the sarcomatoid histology may be better candidates for checkpoint inhibitors [53, 153, 195]. Indeed it may be that PD-L1 negative non-sarcomatoid patients should initially be treated with a chemotherapy regimen and then proceed to a checkpoint inhibitor in the salvage setting upon progression, whilst PD-L1 positive patients should be offered first-line nivolumab/ipilimumab. Overall, it would appear that additional studies will be required to further delineate these issues, and improve our understanding of the immune system as a therapeutic target in MPM. Moreover, many new potential checkpoints have yet to be studied for their therapeutic potential in MPM. All these plus the existing checkpoint inhibitors will require the development of new biomarkers for patient stratification, response and also for predicting or monitoring the emergence of resistance to these agents in MPM patients.

## Data Availability

The datasets analyzed during the current study are available in the following repositories: Oncomine: https://www.oncomine.org/resource/login.html [76] UALCAN: http://ualcan.path.uab.edu/ [78].

## Abbreviations

BTN3: Butyrophilin subfamily 3
CAR: Chimeric antigen receptors
CR: Clinical response
CT: Computed tomography
DC: Dendritic cell
DCR: Disease control rate
DLT: Dose limiting toxicity
ECOG: Eastern Cooperative Oncology Group
EPP: Extra-pleural pneumonectomy
EZH2: Enhancer of Zeste 2
FAP: Fibroblast activation protein
FDA: Food and Drug Administration
GEP: Gene expression profile
GM-CSF: Granulocyte macrophage colony-stimulating factor
HDACi: Histone deacetylase inhibitors
HITOC: Hyperthermic intrathoracic chemoperfusion
HLA: Human leukocyte antigen
HRR: Homologous recombination repair
IFN: Interferon
IL-2: Interleukin-2
LAG-3: Lymphocyte activation gene-3
NSCLC: Non-small cell lung cancer
NK: Natural killer
MET: MET protooncogene
MLSN: Mesothelin
MPM: Malignant pleural mesothelioma
MTD: Maximum tolerated dose
ORR: Overall response rate
OS: Overall survival
PARP: Poly ADP ribose polymerase
PD: Progressive disease
P/D: Pleurectomy with decortication
PET/CT: Positron emission tomography–computed tomography
PFS: Progressive free survival
PR: Partial response
QoL: Quality of life
RECIST: Response evaluation criteria in solid tumors
SBRT: Stereotactic body radiotherapy
SOC: Standard of care
TCGA: The cancer genome atlas
TIM3: T-cell immunoglobulin and mucin-domain containing-3
TLR: Toll-like receptor
TMB: Tumor mutational burden
TNF-α: Tumor necrosis factor alpha
TNFRSF: TNF receptor superfamily
TTF: Tumor treating fields
VATS: Video-assisted thoracoscopic surgery
VEGF: Vascular endothelial growth factor
VISTA: V-type immunoglobulin domain-containing suppressor of T cell activation
